# Dual-Dynamic Covalently Cross-Linked Polyglycerol Hydrogels for Tumor Spheroid Culture

**DOI:** 10.1021/acs.biomac.4c01744

**Published:** 2025-05-10

**Authors:** Jun Feng, Polina Ponomareva, Kunpeng Liu, Chuanxiong Nie, Rui Chen, Rainer Haag

**Affiliations:** Institute of Chemistry and Biochemistry, 9166 Freie Universität Berlin, Takustr. 3, Berlin 14195, Germany

## Abstract

Advancing cancer research depends significantly on developing accurate and reliable models that can replicate the complex tumor microenvironment. Tumor spheroids—three-dimensional clusters of cancer cells—have become crucial tools for this purpose. The overarching goal of tumor spheroid culture is to develop biomaterials that mimic the dynamic mechanical behavior of the native extracellular matrix, enabling high-fidelity culture models. In this study, we developed dynamic hydrogels based on dual-dynamic covalently cross-linked polyglycerol, using boronate bonds and Schiff-base interactions. In addition to good biocompatibility and long-term stability, the hydrogels showed tunable mechanical properties that enabled cells to actively remodel their surrounding microenvironment. This platform was used for successful 3D culture of various cancer cell lines, including HeLa, A549, HT-29, BT-474, and SK-BR-3, which were encapsulated in situ and formed 3D tumor spheroids. These results demonstrate the feasibility and versatility of our dynamic hydrogel system in supporting tumor spheroid culture.

## Graphical abstract

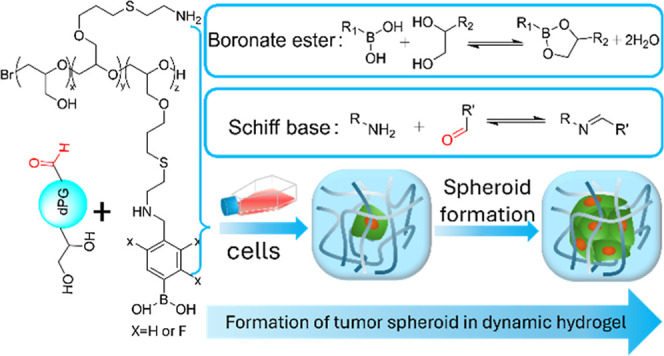

## Introduction

Cancer research, in both academia and the pharmaceutical industry, has traditionally used various in vitro cell-based models to study the signaling pathways and mechanisms driving cancer cell behaviors, such as metabolism, growth, migration, matrix invasion, and drug resistance.^1,2^ Progress in the field depends on the development of accurate and reliable models that can replicate the complex tumor microenvironment.^3^ In this context, tumor spheroids—three-dimensional clusters of cancer cells—have become essential tools. Unlike conventional two-dimensional cell cultures, spheroids better mimic the architecture, cellular diversity, and nutrient gradients found in living tumors.^4–6^ This makes them a more realistic platform for studying cancer biology, assessing drug responses, and understanding therapeutic resistance. Therefore, cultivating tumor spheroids is crucial for advancing our knowledge of cancer progression and treatment effectiveness in order to bridge the gap between in vitro experiments and clinical outcomes.^3,5,6^

The selection of biomaterials for culturing tumor spheroids is a critical factor in ensuring the success and reliability of these models. Biomaterials provide the structural and biochemical cues necessary for cells to organize into spheroids while maintaining their viability and functionality. Traditionally, natural hydrogels such as matrigel and collagen are often used because they provide a matrix that resembles the extracellular environment found in tissues. These materials support cell adhesion and promote cell growth, maintaining a similar composition to the native extracellular matrix (ECM), which is vital for proper cellular function and organization.^6^ However, natural hydrogels suffer from batch-to-batch variability and may lack tunable properties, making synthetic hydrogels an attractive alternative. Synthetic hydrogels, like polyethylene glycol (PEG),^7^ modified alginates,^8^ engineered supramolecular hydrogels,^9^ offer the advantage of adjustable mechanical and biochemical properties, which can be precisely controlled to mimic various aspects of the tumor microenvironment, such as stiffness, porosity, and biochemical composition.^6^ This flexibility allows researchers to better replicate the conditions found in different types of tumors, facilitating the study of cancer cell behavior under diverse conditions.

In recent years, dynamic covalent hydrogels have emerged as a promising class of materials for 3D cell culture applications, including tumor spheroid formation.^10^ Their appeal lies in the use of dynamic covalent chemistry for cross-linking, which creates hydrogels that mimic the viscoelastic behavior of human tissues such as the brain, liver, muscle, and adipose tissues.^11^ The key feature of dynamic covalent bonds is their reversibility, allowing them to break and reform over time scales that are relevant to cell-driven matrix remodeling, an important aspect for 3D cultures.^12^ These bonds endow hydrogels with unique properties like self-healing, stimuli-responsiveness, and adjustable mechanical strength, making them well-suited for replicating the dynamic nature of the tumor microenvironment.^12^ For instance, the self-healing ability enables the hydrogel to restore its structure after mechanical disruptions, which is advantageous for long-term culture and handling.^13^ Examples of dynamic covalent bonds used in 3D cell culture include boronate ester,^14–17^ imine (hydrazone) cross-linking,^18–24^ thiol–disulfide exchange,^25–27^ Diels–Alder,^28–32^ and oxime.^33–36^ These methods can be performed under physiological conditions, with fast reaction kinetics that can be tailored to achieve a wide range of viscoelastic properties.

However, the reversible nature of dynamic covalent bonds makes their stability susceptible to environmental factors such as pH, temperature, and the presence of competing molecules.^37^ To address this, most hydrogels incorporate both dynamic and permanent covalent bonds to achieve desirable mechanical strength. On the other hand, dual-dynamic covalent cross-linking, which utilizes two types of reversible bonds, can offer enhanced dynamic properties essential for bioapplications, such as improved self-healing abilities, better biocompatibility, tunable mechanical properties, and responsiveness to multiple stimuli. Despite its potential to significantly strengthen and stabilize hydrogels, making them more suitable for long-term cell culture, this approach remains relatively underutilized.^38^ Here we report on a dual-dynamic covalently cross-linked polyglycerol hydrogel to create a more stable platform for culturing tumor spheroids. By leveraging the benefits of polyglycerol and dual dynamic covalent bonding, this work aims to produce hydrogels that not only better mimic the dynamic viscoelastic properties of the tumor microenvironment but also maintain stability over extended culture periods.

## Materials and Methods

### Materials

All chemicals and solvents were obtained from commercial suppliers and used without further purification unless otherwise stated. Glycidol, allyl glycidyl ether (AGE), Tetraoctylammonium bromide, Irgacure 2959 and NaIO_4_ are purchased from Sigma-Aldrich. Ethyl vinyl ether and dry methanol are bought from Thermo Scientific. Triisobutylaluminum (1.1 M in toluene), cysteamine, 4-formylphenylboronic acid, 2-fluoro-4-formylphenylboronic acid and 3,5-difluoro-4-formylphenylboronic acid are purchased from abcr GmbH. Dry toluene, diethyl ether, methanol, hydrochloric acid, tetrahydrofuran (THF) and NaBH_4_ are obtained from Acros Organics. Deionized water (DI water) was purified using a millipore water purification system, achieving a minimum resistivity of 18.0 MΩ·cm. The average molecular weight of 10 kDa hbPG was prepared following a previously reported method.^39^ AGE was dried by stirring with CaH_2_, then distilled under vacuum before use and stored over molecular sieves. Glycidol was protected by reacting with ethyl vinyl ether to obtain ethoxyethyl glycidyl ether (EEGE) according to a previous report.^40^ EEGE was further purified by stirring with CaH_2_, then distilled under vacuum before use and stored over molecular sieves.

### Synthesis of Linear Polyglycerol-*co*-poly­(allyl Glycidyl Ether) (lPG–AGE)

The starting polymer lPG–AGE was synthesized as previously described, using a different initiator.^41^ In summary, lPG–AGE was synthesized using tetraoctylammonium bromide as the initiator through ring-opening anionic polymerization of acetal-protected glycidyl monomers (EEGE) and allyl glycidyl ether (AGE). This was followed by acetal deprotection under acidic conditions in THF (Figure 1a). For the experiments, three variants of lPG–AGE with different numbers of AGE units in the polymer chains were synthesized: lPG-15AGE, lPG-20AGE, and lPG-25AGE. ^1^H NMR of lPG-AGE (MeOD) is shown in Figure S1c. GPC results can be found in Figure S1a.

**Figure 1:**
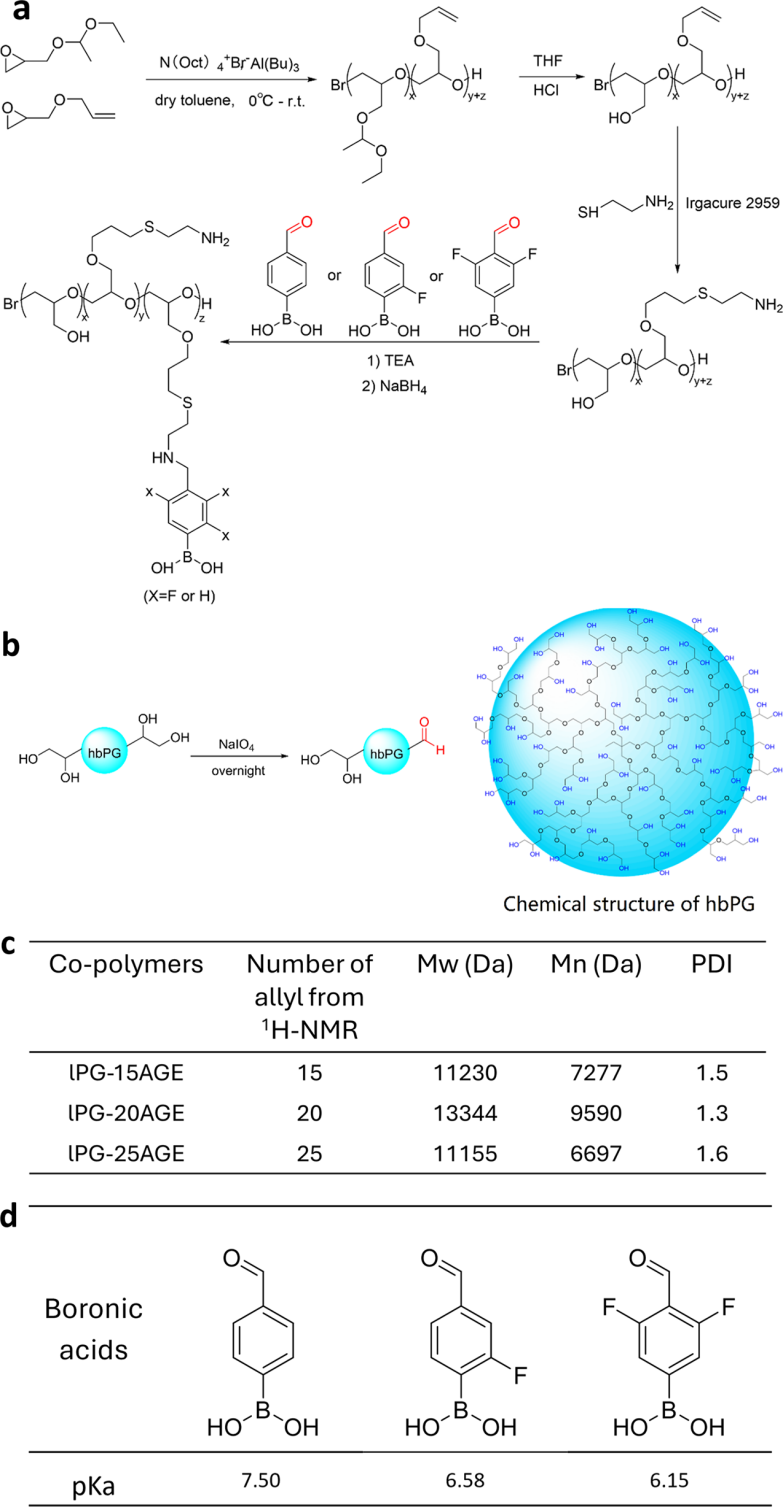
(a) Schematic illustration of the synthesis process for lPG functionalized with amine and phenylboronic acid groups. (b) Schematic illustration of the oxidation of hbPG to produce aldehyde-modified hbPG, along with an idealized structure of hbPG. (c) Molecular weight (Mw), number-average molecular weight (Mn), and dispersity (PDI) of lPG-AGEs, as well as the number of allyl groups on the copolymers, determined from GPC and ^1^H NMR measurements. (d) The p*K*_a_ values of various phenylboronic acid monomers.

### Synthesis of Amine Coupled lPG (lPG-Amine)

Amine groups were coupled to lPG-AGE through the Thiol-Ene click reaction. For instance, 1 g (1 equiv) of lPG-20AGE was dissolved in 50 mL of water, followed by the addition of cysteamine (463 mg, 60 equiv) and the photoinitiator Irgacure 2959 (50 mg). To remove oxygen, the solution was bubbled with nitrogen (N_2_) for 20 min. The reaction was then carried out under UV irradiation for 1 h. After the reaction, the mixture was transferred to a dialysis tube (MW cutoff: 2 kDa) and dialyzed against DI water for 3 days, with the water being changed 3 times per day. The product was obtained by lyophilization. ^1^H NMR of lPG-Amine (MeOD) is shown in Figure S1d.

### Synthesis of Phenylboronic Acid Modified lPG (lPG-Bor)

This procedure aimed to achieve the desired functionalization of lPG-Amine (with 20 amine groups, 1 equiv). One gram of lPG-Amine was dissolved in dry methanol at a concentration of 25 mg/mL, followed by the addition of 4-formylphenylboronic acid (157.4 mg, 10.5 equiv). The solution was bubbled with nitrogen for 20 min to remove oxygen. Then, triethylamine (TEA, 558 μL, 40 equiv) was added with a syringe. After stirring for 2 days, sodium borohydride (NaBH_4_, 75.7 mg, 20 equiv) was slowly added to the reaction mixture, avoiding excessive bubble formation. Stirring continued overnight. The product was purified by dialysis (MW cutoff: 2 kDa). Methanol was used as the solvent for the first three exchanges, followed by water for four to five exchanges. The product was obtained by lyophilization. Different phenylboronic acids (with different numbers of fluorine on phenyl) modified lPG were obtained in the same method. ^1^H NMR results of lPG-Bor (D_2_O) are shown in Figure S1e,f.

### Synthesis of hbPG-CHO

hbPG-CHO was synthesized by oxidizing hyperbranched polyglycerol. The diol groups in hbPG can be oxidized into functional aldehyde groups using NaIO_4_ (Figure 1b). The degree of oxidation was carefully controlled by adjusting the amount of NaIO_4_, in molar equivalents, to achieve the desired level of oxidation. For example, hbPG–CHO–20 was synthesized by adding NaIO_4_ (427.8 mg, 20 equiv) to a flask containing dissolved hbPG (1 g, 1 equiv) in 50 mL of water, and the mixture was stirred overnight. The flask was completely covered with aluminum foil to prevent unwanted light exposure. Upon completion of the reaction, impurities were removed by dialysis against water (MW cutoff: 2 kDa) for 2 days under constant stirring. The purified polymer was then lyophilized to obtain solid hbPG-CHO. By changing the molar equivalents, hbPG–CHO–10, hbPG–CHO–15, hbPG–CHO–20 and hbPG–CHO–25 were synthesized. The FTIR and ^1^H NMR results of hbPG-CHOs are shown in Figure S2.

### Hydrogel Formation

To form the hydrogel, solutions of lPG-Bor and hbPG-CHO in phosphate-buffered saline (PBS) with different concentrations were prepared. Subsequently, equal volumes of lPG-Bor and hbPG-CHO at different concentrations were mixed.

### Cell Culture

All cell lines were obtained from the DSMZ (German Collection of Microorganisms and Cell Cultures GmbH, Braunschweig, Germany) and cultured in Dulbecco’s Modified Eagle Medium (DMEM, high glucose, GlutaMAX, Gibco). The medium was supplemented with 10% (v/v) fetal bovine serum (FBS, Gibco) and 1% (v/v) penicillin–streptomycin solution (Gibco). Cells were maintained at 37 °C in a culture flask with 5% CO_2_.

### Tumor Spheroid Formation

Cells were suspended in DMEM (Dulbecco’s Modified Eagle Medium) at a concentration of 4 × 10^5^ cells/mL. This suspension was then mixed with an equal volume of 5 wt % hbPG–CHO–20 (in DMEM), resulting in a final cell concentration of 2 × 10^5^ cells/mL and a hbPG–CHO–20 concentration of 2.5 wt %. Hydrogels, with a volume of 80 μL, were formed in 96-well plates or 18-well μ-Slides (ibidi, Gräfelfing, Germany) by combining equal volumes of the cell-containing hbPG–CHO–20 solution and 10 wt % lPG-10Bor-1F (in PBS buffer). After incubating at 37 °C for 30 min, the hydrogel networks were fully formed. Subsequently, 200 μL of DMEM medium was added to each well containing hydrogels. The hydrogels were incubated at 37 °C with 5% CO_2_, and the medium was replaced every other day. HeLa-GFP cells (HeLa cells expressing green fluorescent protein) were used to study tumor spheroid growth. During this process, green fluorescent protein (GFP) and bright-field (BF) images were captured using a Zeiss Axio Observer Z1 microscope to monitor the cells and spheroids.

### Statistical Analysis

The quantified data are expressed as mean ± SD. One-Way ANOVA in origin was employed for statistical analysis.

## Results and Discussion

### Synthesis of Hydrogel Precursors

In this study, two types of dynamic covalent chemistries—boronate bond and Schiff base—were used to develop dynamic hydrogels for 3D cell culture. Linear polyglycerol (lPG) was functionalized with amine and various phenylboronic acid groups, while hyperbranched polyglycerol (hbPG) was partially oxidized to introduce aldehyde groups. The free amine groups on lPG reacted with the aldehyde groups on hbPG to form Schiff-bases, while the phenylboronic acid groups interacted with the diols on hbPG to form boronate bonds. Together, these interactions led to the formation of dynamic hydrogels.

### Synthesis of Amine and Phenylboronic Acid Groups Functionalized lPG

The starting material, lPG–AGE, was synthesized as described previously (Figure 1a).^41,42^ The free allyl groups on lPG–AGE enable easy amine coupling and subsequent functionalization with phenylboronic acid groups. Three lPG-AGE variants, each with different numbers of AGE units in the polymer chain, were synthesized: lPG-15AGE, lPG-20AGE, and lPG-25AGE. The molecular weights of the synthesized copolymers, determined by gel permeation chromatography (GPC) (Figures 1c and S1a), were 11.2 kDa, 13.3 kDa and 11.2 kDa for lPG-15AGE, lPG-20AGE, and lPG-25AGE, respectively. These values slightly deviate from the expected molecular weights (11.8 kDa, 12.4 kDa and 13.0 kDa), which can be attributed to experimental variations and measurement errors. However, as confirmed by ^1^H NMR (Figures 1c and S1c), the average number of allyl groups in the synthesized copolymers increased as expected. The stoichiometrically controlled allyl group content in polymer chains allows for precise control of functionalization in subsequent steps.

Next, amine groups were introduced to the starting material via a UV-assisted thiol–ene click reaction between the allyl groups on lPG-AGE and the thiol groups on cysteamine (Figure 1a). The ^1^H NMR spectra (Figure S1d) confirm the complete consumption of allyl groups. All peaks corresponding to the protons on carbon–carbon double bonds of allyl groups (6.0–5.1 ppm) disappeared, indicating full conversion. After the coupling reaction, four new peaks appeared in the 3.2–1.7 ppm range, which can be attributed to protons on carbons adjacent to sulfur. Additionally, due to the consumption of allyl groups and the coupling of cysteamine, the peaks originally at 4.1–4.0 ppm shifted to 3.85–3.5 ppm, overlapping with the backbone signals. The disappearance of double bond protons and the emergence of new peaks confirm the successful coupling of cysteamine. Furthermore, integration of the spectra indicates 100% conversion of the reaction. The amine groups served as sites for coupling phenylboronic acid groups. The resulting polymers are designated as lPG-15Amine, lPG-20Amine and lPG-25Amine.

Phenylboronic acid groups were attached through Schiff-base formation between the amine groups on lPG and the aldehyde group of phenylboronic acid (Figure 1a). However, since Schiff bases are unstable and can undergo reversible hydrolysis,^43,44^ NaBH_4_ was used to reduce the imine bonds to stable amines after the reaction, ensuring that the phenylboronic acids were securely attached to lPG.

Two factors influence the boronate bond-based network: the type of phenylboronic acid and the number of boronate bonds. Electron-withdrawing substituents on the aromatic ring of phenylboronic acid lower the p*K*_a_, enhancing the reaction between phenylboronic acid and diols.^14,45^ To explore this effect in our dynamic hydrogel, three phenylboronic acid monomers with varying numbers of fluorine (F) atoms on the aromatic ring were used to functionalize lPG. As shown in Figure 1d (obtained from Figure S1b), the p*K*_a_ values decreased as the number of electron-withdrawing substituents (F atoms) increased. To investigate the effect of these different phenylboronic acids on the mechanical properties of dynamic hydrogels, lPG-20Amine was selected as the base material for coupling with the three monomers. In the ^1^H NMR spectra (Figure S1e), new peaks appearing in the 8.0–6.8 ppm range correspond to protons on the phenyl ring, confirming the successful coupling of phenylboronic acid. Additionally, peak integration analysis indicates that, on average, approximately 10 phenylboronic acid groups are attached to each polymer chain.

To control the number of boronate bonds, three differently functionalized lPG polymers—containing 5, 10, and 15 phenylboronic acid groups (of the same phenylboronic acid species)—were synthesized from lPG-15Amine, lPG-20Amine, and lPG-25Amine, respectively. In the ^1^H NMR spectra (Figure S1f), new peaks in the 7.5–7.0 ppm range confirmed the successful coupling of phenylboronic acid. And the peak integration increased with the feed ratio of phenylboronic acid, aligning well with expectations. Apart from the phenylboronic acid groups, these three polymers had the same number of amine residues, ensuring that the effect of the Schiff base was consistent across the samples.

### Synthesis of Aldehyde Functionalized hbPG (hbPG-CHO)

The chemical structure of hbPG (Figure 1b) reveals a high concentration of diols, which can be oxidized into functional aldehyde groups using NaIO_4_. FTIR spectroscopy confirmed the presence of aldehyde carbonyl bonds, indicating the successful oxidation of hbPG (Figure S2a). Compared to hbPG, hbPG-CHO exhibits weaker OH stretching modes (3650 cm^–1^–3050 cm^–1^) due to the consumption of hydroxyl groups during oxidation. A small peak at 1731 cm^–1^ corresponds to aldehyde groups, which form intramolecular hemiacetal structures, a common occurrence in dry polymers containing aldehyde groups.^46^ ^,^ ^47^ ^1^H NMR spectroscopy was conducted to further confirm the oxidation of hbPG. However, the characteristic proton signal of the aldehyde group was not observed in the spectrum, likely due to the formation of hydrate species. Although aldehyde protons are not exchangeable like −OH or −NH protons, they can still react with water, especially in aqueous environments. In such cases, aldehydes can exist in equilibrium with their geminal diol (hydrate) form, which lacks the aldehyde proton. As a result, the corresponding NMR signal can disappear or become significantly weakened. Nevertheless, the oxidation of hbPG can be indirectly verified by the appearance of characteristic signals from protons on carbon atoms adjacent to the aldehyde group (Figure S2b, indicated by red arrows). The degree of oxidation can be conveniently adjusted by varying the molar ratio of NaIO_4_, allowing for control over the oxidation level. In this study, NaIO_4_ was added at molar feed ratios of 10:1, 15:1, 20:1, and 25:1 relative to hbPG. The resulting products are referred to as hbPG–CHO–10, hbPG–CHO–15, hbPG–CHO–20, and hbPG–CHO–25, respectively. As shown in Figure S2b, the signal integrations increased with higher NaIO_4_ feed ratios, indicating increased oxidation. However, the integration values were lower than expected, suggesting that oxidation may not have occurred exclusively at the diol groups. NaIO_4_ may also oxidize other sites within the hbPG structure, as supported by the appearance of new peaks at 4.35 and 3.15 ppm (green arrows in Figure S2b). Based on the signal integration, the conversion of oxidation to aldehyde groups was calculated to be approximately 83.13 ± 4.30%. By using hbPG samples with different degrees of oxidation, this study is able to investigate how aldehyde concentration influences the mechanical properties of the resulting hydrogels.

### Formation and Mechanical Properties of Dynamic Hydrogels

Boronate bonds and Schiff-bases are widely used dynamic covalent chemistries for developing dynamic hydrogels. Boronate bonds are often formed through the interaction between boronic acids and *cis*-1,2-diols. Compared to other reversible bonds, boronates exhibit much faster association and dissociation dynamics.^14,48^ A Schiff base is typically formed through the condensation of primary amines and active carbonyl groups via nucleophilic addition, producing a hemiaminal. This intermediate then undergoes dehydration to generate an imine bond,^49^ which is a reversible dynamic covalent bond that exhibits fast stress relaxation.^50^ In this study, hydrogels were formed using both of these dynamic covalent bonds (Figure 2a).

**Figure 2:**
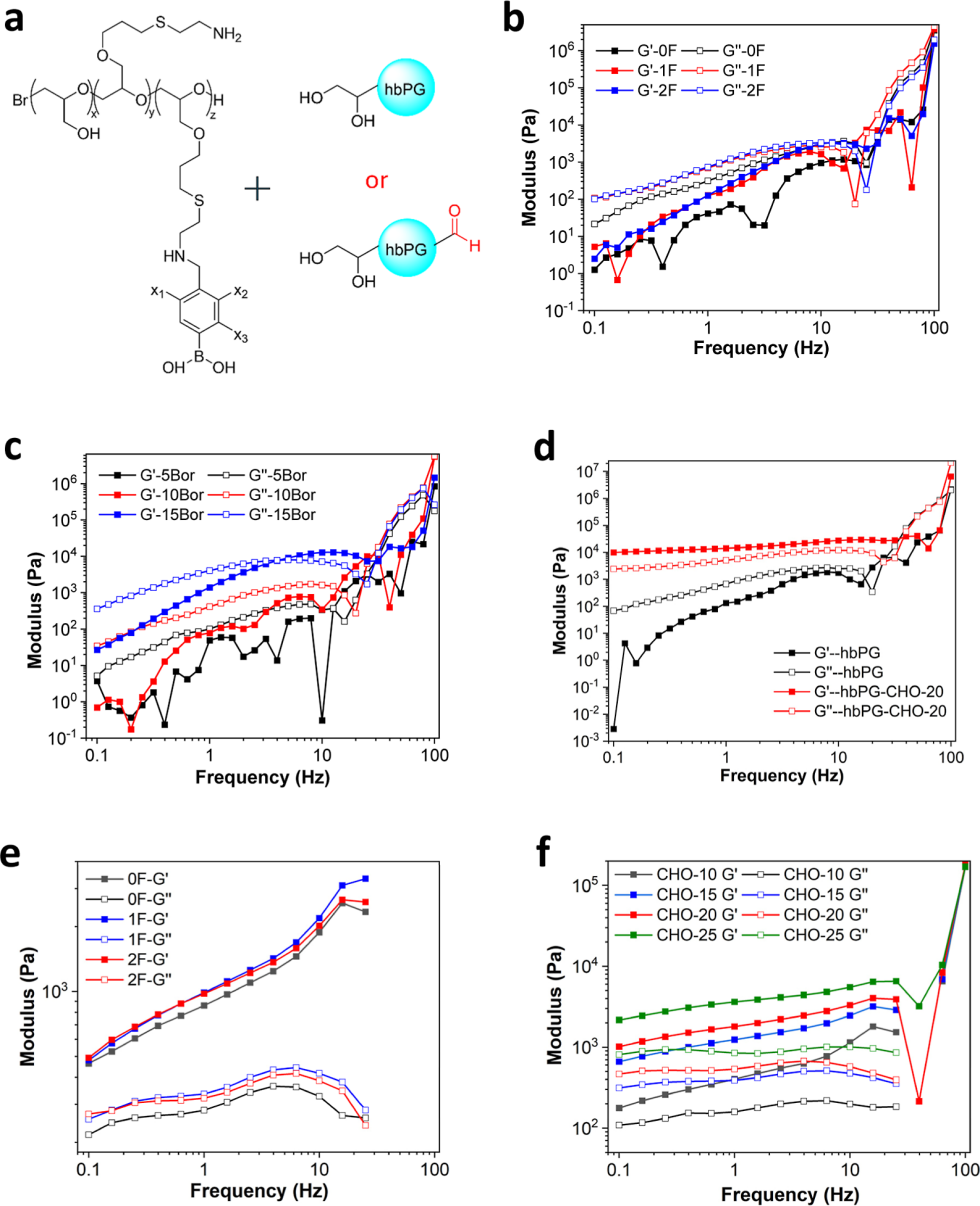
(a) Chemical structures of dynamic hydrogel precursors. (b) Shear modulus (*G*′ and *G*″) of mixtures composed of hbPG and various types of phenylboronic acid-functionalized lPG, each differing in the number of fluorine atoms on the aromatic ring. (c) Shear modulus (*G*′ and *G*″) of mixtures consisting of hbPG and lPG functionalized with different amounts of 2-fluorophenylboronic acid. (d) Shear modulus (*G*′ and *G*″) of mixtures prepared from 2-fluorophenylboronic acid-functionalized lPG and either hbPG or hbPG–CHO–20. (e) Shear modulus (*G*′ and *G*″) of mixtures formed from hbPG–CHO–15 and various phenylboronic acid-functionalized lPG types, with differing numbers of fluorine atoms on the aromatic ring. (f) Shear modulus (*G*′ and *G*″) of hydrogels consisting of 2-fluorophenylboronic acid-functionalized lPG and hbPG-CHO with varying degrees of oxidation. All shear modulus measurements were obtained through frequency sweep tests conducted at 1% strain and room temperature.

Before forming a hydrogel via boronate and Schiff-base chemistry, we investigated whether hbPG-CHO undergoes self-gelation due to interactions between aldehyde and hydroxyl groups. To assess this, we performed a time sweep test (at a fixed strain and frequency) using a rheometer to analyze the behavior of DMEM (cell culture medium), hbPG–CHO–20 solution, hbPG, and a mixture of hbPG-CHO and hbPG (all polymers were dissolved in DMEM). As shown in Figure S3a, all polymer solutions exhibited a higher storage modulus than DMEM, likely due to the high viscosity of the polymers. However, there was almost no difference among the polymer solutions, suggesting that the interaction between aldehyde and hydroxyl groups is too weak to be detected. Therefore, we assume that this interaction contributes little to the mechanical properties of the hydrogel.

Since both boronate and Schiff-base formation are highly pH-dependent, we examined the gelation behavior of hydrogels at different pH levels. The initial pH of the lPG-Bor solution was approximately 9, while the hbPG solution had a pH of around 7.4. Mixing these two solutions resulted in a pH of approximately 8.5. To investigate the effect of pH, we adjusted the pH of both solutions to 5, 6, 7, and 9.5. The gelation behavior of the mixtures was then tested at pH 5, 6, 7, 8.5, and 9.5.

Under acidic and neutral conditions, no gelation was observed, likely due to reduced boronic acid ionization and the protonation of amines. In contrast, hydrogel formation was successful under basic conditions, with higher pH levels facilitating gelation (Figure S3b). Additionally, the mechanical properties of hydrogels formed at pH 9.5 were higher than those formed at pH 8.5. However, considering the importance of biocompatibility in biological experiments, hydrogels formed at pH 8.5 were chosen for further studies, as this pH is closer to physiological conditions.

We then investigated how introducing F atoms onto the aromatic ring of phenylboronic acid influences the formation of boronate bonds. We prepared 10 wt/v % solutions of phenylboronic acid, 2-fluorophenylboronic acid, and 3,5-difluorophenylboronic acid-modified lPGs (each with 10 boronic acid groups per lPG) and mixed each solution separately with a 10 wt/v % solution of unmodified hbPG in equal volume. Upon mixing, all solutions rapidly became highly viscous, and we analyzed the mechanical properties of the mixtures using rheological measurements. As shown in Figure 2b, the introduction of F atoms on the aromatic ring enhanced the mechanical strength, indicated by increases in both *G*′ and *G*″. However, there were no significant differences between the mixtures of 2-fluorophenylboronic acid and 3,5-difluorophenylboronic acid-modified lPGs. This can be attributed to the different positions of the F atoms on the aromatic ring, which may weaken the electron-withdrawing effect as the substituents move further from the boronic acid group.

Moreover, Figure 2b shows that *G*″ values were higher than *G*′, indicating that the mixtures remained in a liquid state. This is likely due to two factors: (1) boronate bonds exhibit fast association and dissociation dynamics,^48^ leading to weak bond strength, insufficient for solidification, and (2) the number of boronate bonds is not enough to form a solid hydrogel.

To assess whether increasing the number of boronate bonds could shift the mixture from liquid to solid, we mixed 10 wt/v % solutions of 2-fluorophenylboronic acid-modified lPGs (with 5, 10, and 15 boronic acid groups per lPG) with 10 wt/v % unmodified hbPG in equal volume. Similarly, all solutions rapidly became highly viscous upon mixing. Although both *G*′ and *G*″ increased with the number of 2-fluorophenylboronic acid groups, all samples remained in a liquid state, as indicated by *G*″ being higher than *G*′ (Figure 2c). This suggests that boronate bond cross-linking alone among the polymers (lPG) is not strong enough to support a stable hydrogel network.

Next, we introduced a second dynamic covalent bond—Schiff base cross-linking—into the system. We anticipated the introduction of the second dynamic covalent linkage would increase the cross-linking efficiency of polymers and result in hydrogel formation. For comparison, two mixtures were prepared: one consisting of a 10 wt/v % solution of 2-fluorophenylboronic acid-modified lPG (with 10 boronic acid groups per lPG) mixed with 10 wt/v % unmodified hbPG, and another with the same lPG mixed with 10 wt/v % hbPG–CHO–20. As shown in Figure 2d, the dual cross-linked mixture exhibited solid-like behavior, as indicated by *G*′ being higher than *G*″. Additionally, both *G*′ and *G*″ increased by 2 orders of magnitude after introducing the Schiff base cross-linking network. The enhanced mechanical properties result not only from the formation of the dual cross-linked network but also from the mutual facilitation between the two dynamic covalent bonds. Boronic acids, as Lewis acids, can interact with electron-rich species. Specifically, they coordinate with the carbonyl oxygen of aldehydes, increasing the electrophilicity of the carbonyl carbon, which promotes nucleophilic attack by amines to form C = N imine bonds. Conversely, amines can interact with the electron-deficient boron center via N→B dative (coordinate covalent) bonds, enhancing the Lewis acidity of boron and making it more reactive toward diols. Therefore, the resulting hydrogel system is not merely a combination of two independent dynamic networks, but a synergistic dual cross-linking system where both types of bonds mutually enhance and stabilize each other. To investigate the effect of F on the mechanical properties of dual dynamic covalently cross-linked hydrogels, we tested the mechanical properties of hydrogels formed by mixing solutions of various phenylboronic acid-modified lPGs with hbPG–CHO–15. As shown in Figure 2e, varying the types of phenylboronic acids had little effect on the hydrogels’ mechanical properties.

We then investigated the effect of Schiff-base cross-linking density on mechanical properties of hydrogels. We synthesized hbPGs with four different oxidation levels and mixed them with 2-fluorophenylboronic acid-modified lPG (with 10 boronic acid per lPG) at 10 wt/v %. The mechanical properties of the hydrogels consistently increase with higher oxidation levels of hbPG (Figure 2f), despite the limited number of available imine bonds due to only 10 NH_2_ groups per lPG. Higher oxidation levels provide a greater concentration of cross-linking sites, enhancing hydrogel stability. The tunable mechanical properties make the dynamic hydrogel adaptable to different cell types, expanding its potential applications.

In all the frequency sweep tests, fluctuations were observed, particularly in weak hydrogels. This phenomenon can be attributed to the dynamic nature of the cross-linked network. In our hydrogels, the network is formed by boronate and Schiff-base bonds, both of which are dynamically covalent cross-linking systems. These reversible bonds continuously break and reform under stress. During oscillatory shear in a frequency sweep test, some bonds dissociate and reassociate at different time scales, leading to temporary softening or stiffening of the network. Additionally, applied shear stress can momentarily disrupt weak cross-links, and as the stress relaxes, the bonds reform, resulting in time-dependent variations in mechanical properties. The frequency of the applied deformation may either align with or interfere with the bond exchange kinetics, further contributing to fluctuations. Moreover, the rapid formation of boronate bonds may lead to an uneven distribution of cross-linking density, where localized regions experience temporary softening due to bond dissociation, causing variations in the measured stiffness. Ultimately, the dynamic behavior of these two reversible bonds, along with the potential heterogeneity in the cross-linking network, may be responsible for the observed fluctuations.

### Shear-Thinning and Self-Healing Properties of Dynamic Hydrogels

In this study, we aimed to develop a dynamic hydrogel for culturing 3D tumor spheroids. The hydrogels were formed through two types of dynamic covalent bonds—boronate and Schiff-base—which are reversibly degradable. To characterize the dynamic properties of these hydrogels, we conducted shear-thinning and self-healing tests.

Shear-thinning behavior, which is often linked to reversible structural changes in response to mechanical forces, can indicate the disruption and realignment of polymer chains within a hydrogel matrix. As the shear rate increases, the hydrogel network temporarily disentangles, allowing for easier material flow. As shown in Figure S3c, the viscosities of our hydrogels—both with and without fluorine substituents on the aromatic ring of phenylboronic acid—decreased sharply with increasing shear rates. This behavior suggests that our hydrogels can provide a dynamic environment suitable for cell culture.

To further confirm that the cross-linked network can rearrange in response to mechanical forces, we performed a self-healing test. A rheological recovery experiment was conducted to assess the self-healing properties of the hydrogels. As shown in Figure S3e, both *G*′ and *G*″ decreased sharply when the shear strain increased from 1% to 200%, indicating a breakdown of the hydrogel network at high strain. However, when the strain was reduced back to 1%, *G*′ and *G*″ quickly recovered to their original levels, demonstrating the self-healing ability of the hydrogels due to the dynamic covalent bonds. Repeated cycles of the recovery experiment showed consistent results, indicating that the hydrogel network can reform once the applied mechanical force is removed. This self-healing behavior was also visibly evident, as two separate halves of the hydrogel could be seen successfully merging into a single contiguous piece (Figure S3f).

This dynamic behavior of the covalent bonds allows the hydrogel to recover after network disruption, creating a dynamic 3D culture environment in which cells can rearrange their microenvironment.

### Stability of Dynamic Hydrogels

Typical synthetic materials tend to exhibit swelling-induced weakening, in which swelling dilutes the network and leads to a sharp reduction in mechanical strength.^51^ This issue is especially critical in biomedical applications, such as scaffolds for organ or tissue regeneration in tissue engineering. Swelling in body fluids can not only weaken the hydrogel but also compress or even damage surrounding organs or tissues.^52–58^ Therefore, developing antiswelling or low-swelling hydrogels can be advantageous for 3D cell cultures by helping to maintain a stable macroscopic physiochemical environment for cells. Accordingly, we measured the swelling ratio of our dynamic hydrogels. As shown in Figure S4a, the swelling ratio of our hydrogels increased rapidly during the initial immersion in the cell culture medium (Dulbecco’s Modified Eagle Medium-DMEM), reaching equilibrium after approximately 20 min. Notably, the maximum swelling ratio was only about 35%, which is significantly lower than other hydrogels.^59,60^ This low swelling behavior may be attributed to the formation of hydrophobic imine bonds during hydrogel preparation. Consequently, our dynamic hydrogels are capable of providing a stable macroscopic environment for 3D cell culture.

To further assess the long-term stability of the dynamic hydrogels, we conducted a degradability test in the cell culture medium (DMEM). The hydrogels were immersed in DMEM at 37 °C, and their mechanical properties were analyzed through rheological experiments at regular intervals. As shown in Figure S4b, both *G*′ and *G*″ values decreased over time with prolonged immersion in DMEM. Over the 40 day experimental period, *G*′ decreased by approximately 50%, while *G*″ decreased by about 90%. There are two main reasons for the reduction in mechanical strength over time. First, boronic acids are prone to hydrolysis, especially at neutral to basic pH. Since the hydrogels were formed under slightly basic conditions, some boronic acids may have undergone hydrolysis. Second, aldehydes are easily oxidized into carboxyl groups, which cannot form imine bonds with amines. Both factors reduce the density of cross-linking points, ultimately leading to a decrease in mechanical strength. Despite these reductions, the hydrogels retained sufficient mechanical strength to support cell culture. This indicates that our dynamic hydrogels not only allow cells to rearrange their microenvironment during culturing but are also stable enough for long-term cell culture applications.

### Application of Dynamic Hydrogels in Culturing 3D Tumor Spheroids

Hydrogels’ facile tunability makes them an ideal choice for replicating the mechanical properties of the ECM to study cell–environment interactions in ex-vivo models.^61^ Hydrogels cross-linked via dynamic covalent chemistry exhibit viscoelastic behavior similar to human tissues, such as the brain, liver, muscle, and adipose tissues.^11^ Dynamic covalent chemistry enables 3D cell culture systems to mimic the dynamic mechanical behavior of the native ECM, thereby enhancing the accuracy of cell culture models. These dynamic covalent bonds are reversible, allowing them to break and reform over time scales relevant to cell-driven matrix remodeling–a crucial attribute for 3D culture systems.^12^

Dynamic covalently cross-linked hydrogels have been widely used to create stable 3D cell culture models and high-resolution constructs for both in vitro and in vivo applications.^10^ To demonstrate the potential of our dynamic hydrogel in ECM-mimicking applications, we used it as a 3D scaffold for forming tumor spheroids. Using hydrogels as a platform for 3D culture not only provides cell suspension and nonadherent conditions that promote aggregation into multicellular tumor spheroids, but also enables the controlled expansion of cancer stem cells.^62^ To investigate how the hydrogels support and facilitate cell growth, we encapsulated HeLa-GFP cells (HeLa cells expressing green fluorescent protein) in the dynamic hydrogel by mixing them with hydrogel precursors (Figure 3a), then monitored cell growth and tumor spheroid formation. As observed, the mixture containing the precursors and cells quickly became viscous, providing support to keep the cells suspended and prevent them from sinking. This support ensured that the cells were uniformly distributed in three dimensions within the resulting hydrogel. After incubation for 30 min at 37 °C, a second cross-linking network formed, stabilizing the cells within the hydrogel matrix. The hydrogel was then covered with cell culture medium (DMEM) and the cells were cultured for 9 days, with observations and imaging performed at 1, 2, 3, 7, and 9 days.

**Figure 3:**
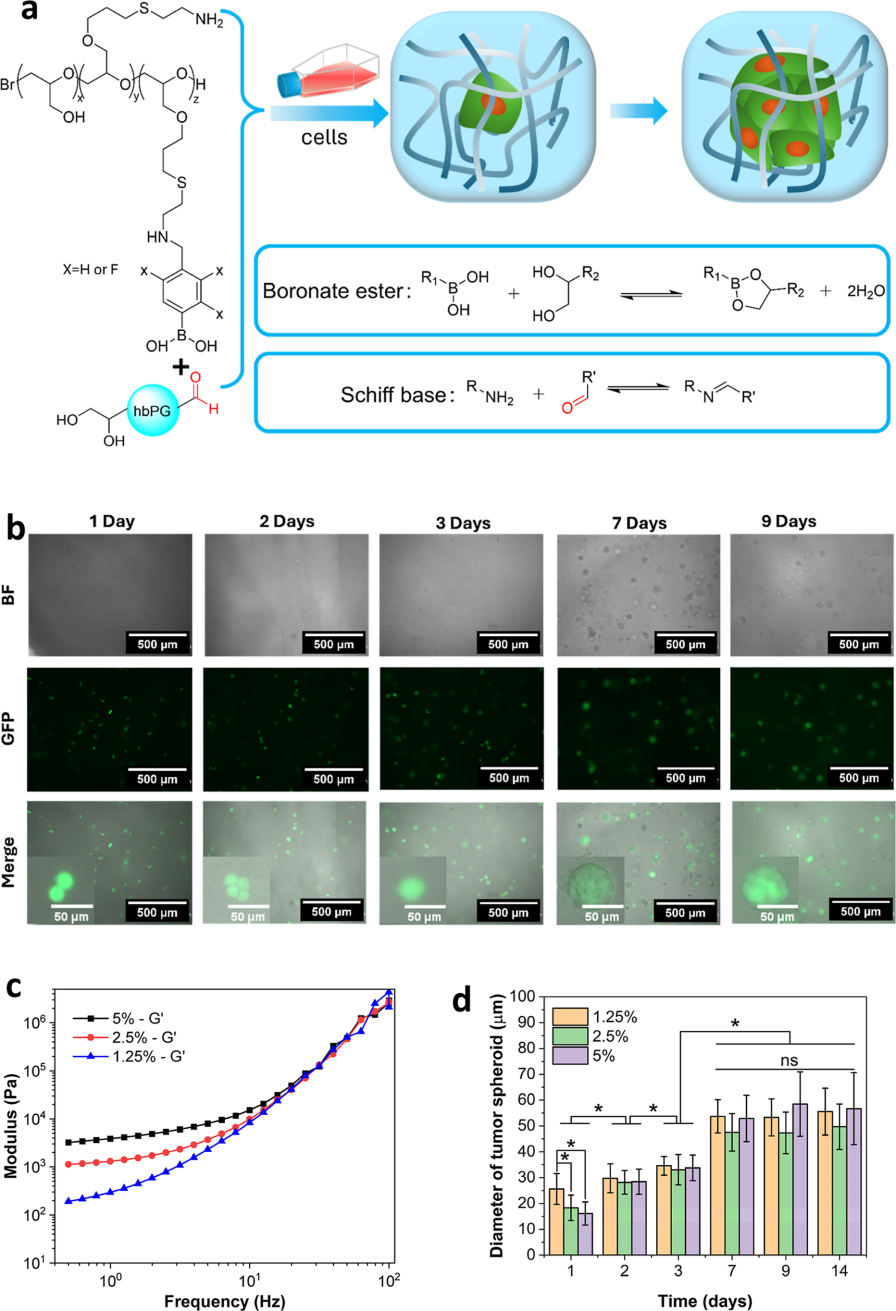

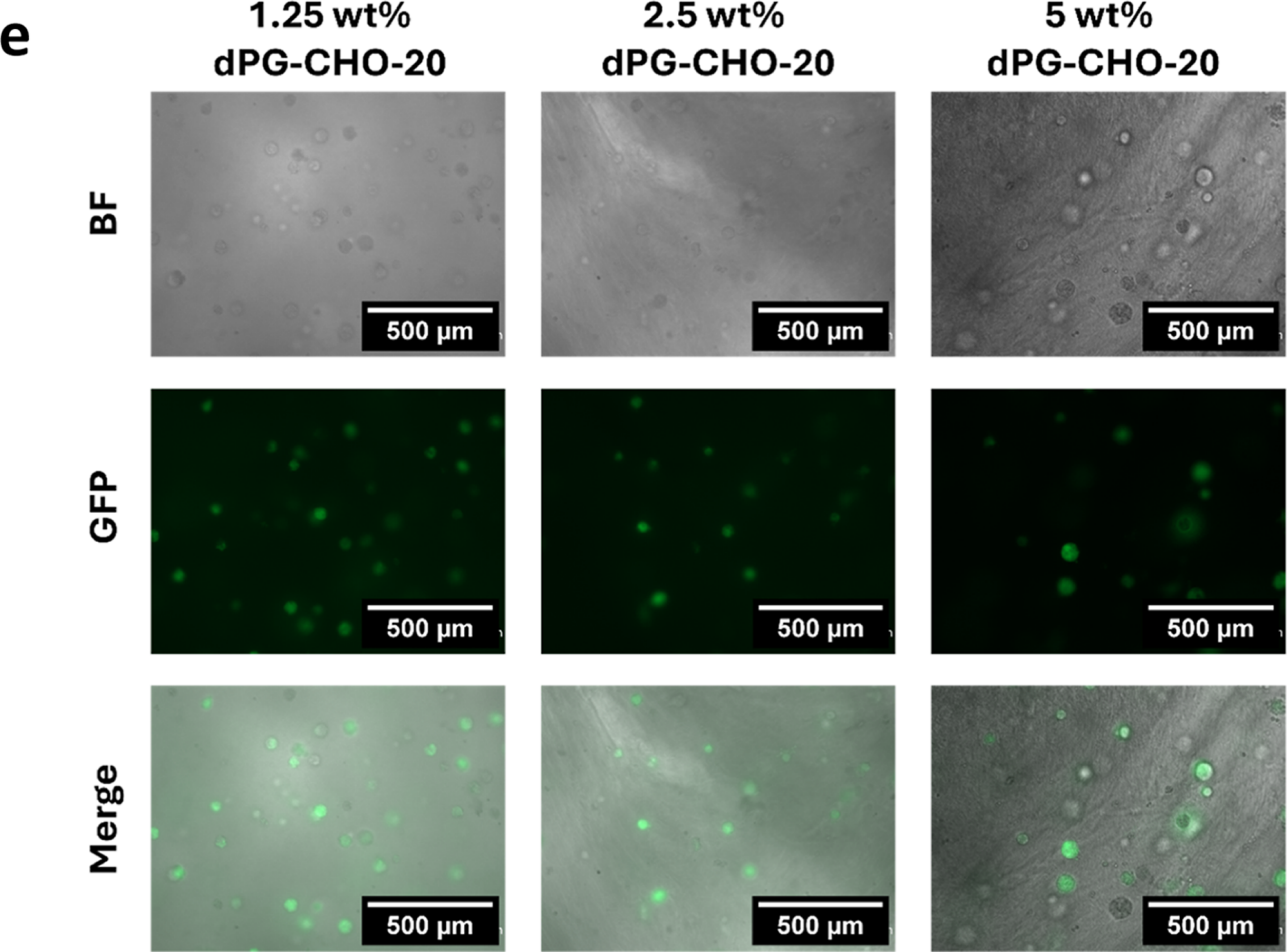
(a) Schematic illustration of cells encapsulation and tumor spheroid formation in dynamic hydrogel dually cross-linked by boronate and Schiff-base bonds. (b) Brightfield and green fluorescence images of HeLa-GFP cells or tumor spheroids in hydrogels composed of 5 wt/v % 2-fluorophenylboronic acid-functionalized lPG and 1.25 wt/v % hbPG–CHO–20 at different time points. (c) Storage modulus (*G*′) of hydrogels made from 5 wt/v % 2-fluorophenylboronic acid-functionalized lPG with varying concentrations of hbPG–CHO–20. (d) Diameters of tumor spheroids grown in hydrogels containing 5 wt/v % 2-fluorophenylboronic acid-functionalized lPG with different concentrations of hbPG–CHO–20. Data is presented as mean ± standard deviation, *n* ≥ 40, * *p* < 0.05, ns denotes no significance *p* > 0.05 (e) Brightfield and green fluorescence images of tumor spheroids cultured for 9 days in dynamic hydrogels made of 5 wt/v % 2-fluorophenylboronic acid-functionalized lPG with different concentrations of hbPG–CHO–20. All concentrations mentioned above refer to the final concentrations in the dynamic hydrogels.

As shown in Figure 3b, after 1 day of culture, all the cells were alive, and most appeared in pairs, indicating that they had completed their first division. Over time, the cells continued to survive, undergoing a second division by day 2, as evidenced by clusters of four cells. By day 3, the divided cells began to merge, forming a tumor spheroid. With extended culture time, the spheroids grew larger, reaching their maximum size after 7 days, with no further growth observed by day 9. The final diameter of the spheroids was approximately 70 μm.

As mentioned above, hydrogels cross-linked via dynamic covalent chemistry exhibit viscoelastic behavior, allowing cells to rearrange their microenvironment accordingly.^11^ Therefore, it is important to investigate how cells and spheroids respond to hydrogels with different mechanical properties. In this study, we used three hydrogels with varying mechanical properties to culture cells and spheroids. These hydrogels were cross-linked using different concentrations of hbPG–CHO–20, resulting in higher storage modulus (*G*′) with increasing concentrations (Figure 3c).

As shown in Figure 3d, tumor spheroids can form and grow in hydrogels with different mechanical properties. However, hydrogel stiffness primarily influences spheroid growth at the early stage. Within the first day, cells proliferate more rapidly in softer hydrogels, suggesting that stiffer hydrogels impose greater physical constraints, leading to smaller initial cluster sizes. From the second day onward, cell proliferation and spheroid growth exhibit similar rates and behavior across all hydrogel conditions. Ultimately, tumor spheroids appear similar in both size and pattern, regardless of the hydrogel’s mechanical properties. This can be attributed to the fact that the hydrogels were cross-linked through two dynamic covalent bonds, allowing cells to remodel their microenvironment to support growth. As a result, the final spheroid sizes were comparable across all hydrogels (Figure 3e). The diameters of cell clusters or spheroids increased over time in all three types of hydrogels. While the size distribution did not show significant differences among the hydrogels, the diameters increased significantly within the first 3 days (Figure 3d). By day 7, tumor spheroid growth had nearly reached saturation. With prolonged culture, spheroids continued to grow slightly, but the differences were not statistically significant.

To demonstrate the versatility of our dynamic hydrogel for culturing tumor spheroids, we used it to encapsulate different cancer cell lines and investigate their ability to form tumor spheroids. Four cell lines were selected for this study: A549 (human lung cancer), BT-474 (ER/HER2-positive human breast cancer), HT-29 (human colorectal cancer), and SK-BR-3 (HER2-positive human breast cancer). Each of these cell lines represents distinct cancer types: A549 models lung cancer, BT-474 and SK-BR-3 represent HER2-positive breast cancer with different receptor profiles, and HT-29 is used as a model for colorectal cancer. These cell lines were chosen to encompass a variety of molecular profiles and cancer origins, allowing us to assess the hydrogel’s ability to support tumor spheroid formation across different cancer types.

To enhance the visualization of the spheroids, the cells were stained with phalloidin and DAPI. Phalloidin is a commonly used stain that binds to actin filaments in the cell’s cytoskeleton, helping to reveal the shape and structure of the cells. DAPI is a fluorescent stain that specifically binds to DNA, allowing visualization of the cell nucleus. When exposed to the appropriate UV light, phalloidin emits red fluorescence, while DAPI emits blue fluorescence, making the cytoskeleton and nucleus easily visible under a microscope. As shown in Figure 4, despite the distinct origins and molecular characteristics of the selected cell lines, all four successfully formed tumor spheroids after 9 days of culture in the dynamic hydrogel. Although different cancer types are expected to exhibit distinct growth patterns,^63–65^ similar tumor spheroids were observed across all cell lines. These results suggest that the dynamic hydrogel is able to support spheroid formation across a wide range of cancer types, highlighting its broad applicability for 3D culture models.

**Figure 4:**
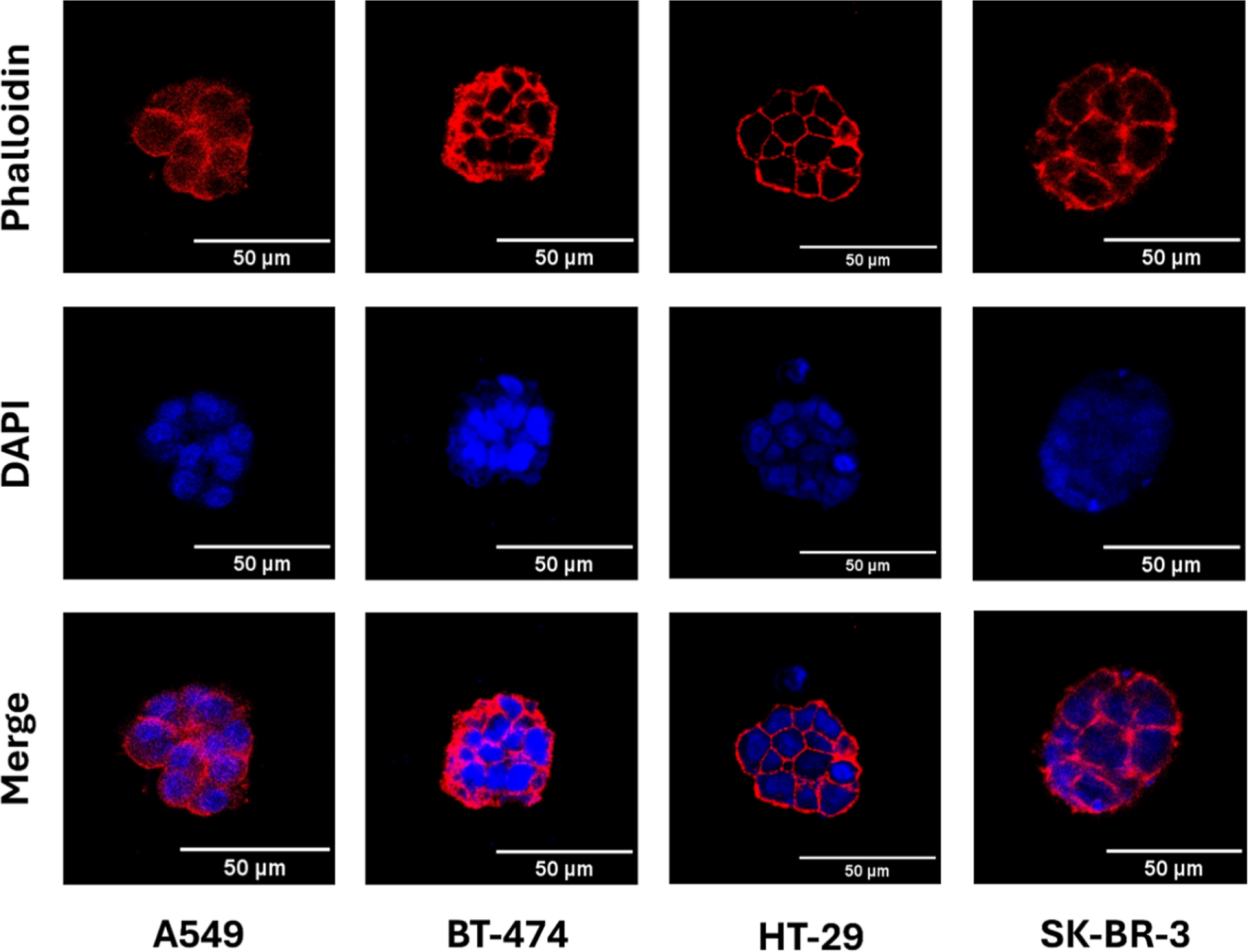
Confocal images of tumor spheroids cultured for 9 days in hydrogels composed of 5 wt/v % 2-fluorophenylboronic acid-functionalized lPG and 1.25 wt/v % hbPG–CHO–20 (all concentrations refer to the final concentrations in the dynamic hydrogels). Cells were stained with phalloidin (red) and DAPI (blue).

Finally, we explored the potential of using the dynamic hydrogel for long-term cell culture. The same four cell lines were encapsulated in the dynamic hydrogels and cultured for 4 weeks. A live/dead assay was conducted to visualize the spheroid morphology and assess cell viability within the spheroids. As shown in Figure S5, all four cell lines formed tumor spheroids, with most of the cells remaining viable. Some dead cells were observed in the centers of the spheroids, which can be attributed to nutrient and oxygen limitations.

Overall, our experiments demonstrate that the dynamic hydrogels exhibit good biocompatibility and adaptable mechanical properties, which support the growth of tumor cells into spheroids. Additionally, the stability of the hydrogels enables long-term cell culture, broadening their potential applications in various fields.

## Conclusions

In summary, we successfully prepared dynamic hydrogels from dual dynamic covalently cross-linked networks, using boronate bonds and Schiff-base interactions. The resulting hydrogels exhibited adjustable mechanical properties, good biocompatibility, and long-term stability. The dual dynamic covalently cross-linked networks imparted reversible mechanical properties to the hydrogels, enabling shear-thinning and self-healing behavior. This reversibility allowed cells to rearrange their microenvironment while encapsulated within the hydrogels. Ultimately, the hydrogels were used for the 3D culture of various cancer cell lines–including HeLa-GFP, A549, HT-29, BT-474, and SK-BR-3–enabling them to grow into tumor spheroids. These cell lines were encapsulated in situ within the gel matrix and subsequently formed 3D tumor spheroids, achieving an average tumoroid size of approximately 70 μm after 7 days of incubation, with no observable toxicity. With its proven ability to maintain its stability for long-term preservation, we believe that this dynamic hydrogel platform has the potential to support not only 3D tumoroid growth but also organoid formation, thereby making this toolkit valuable for 3D cell culture.

## Supplementary Material



## Notes

Experimental details about p*K*_a_ measurement, swelling ratio test, rheological measurements, self-healing tests, in vitro degradation test, staining and imaging of tumor spheroids, and live/dead assay of tumor spheroids; the results of GPC, ^1^H NMR, FT-IR, shear thinning test, self-healing test, swelling ratio test, in vitro degradation test, and live/dead assay of tumor spheroids (PDF)
