# Supplementary material for: Dual-Dynamic Covalently Cross-Linked Polyglycerol Hydrogels for Tumor Spheroid Culture
Source: Biomacromolecules. 2025 May 10;26(6):3331–43. doi: 10.1021/acs.biomac.4c01744 (PMC12152840; doi:10.1021/acs.biomac.4c01744)
Supplement: Supplementary file 1 [file bm4c01744_si_001.pdf]

Supporting Information

Dual-Dynamic Covalently Crosslinked Polyglycerol Hydrogels for Tumor Spheroid Culture

Jun Feng[1], Polina Ponomareva[1], Kunpeng Liu[1], Chuanxiong Nie[1], Rui Chen[1], Rainer Haag[1]**

[1]Institute of Chemistry and Biochemistry, Freie Universität Berlin, Takustr. 3, 14195 Berlin, Germany

*Email: jun.feng@fu-berlin.de and haag@chemie.fu-berlin.de

Instruments and methods

Instruments: All ^{1}H-NMR spectra were recorded at 300 K on a Jeol Eclipse 500 MHz spectrometer (Tokyo, Japan). Chemical shifts (δ) were reported in ppm, with the deuterated solvent peak as a standard. All GPC chromatograms were recorded in water using an Agilent 1100 equipped with an automatic injector, isopump, and differential refractometer (Agilent Technologies, Santa Clara, CA, USA). The PSS Suprema column system (precolumn: 1× with pore size of 30 Å, 2× with pore size of 1000 Å) was calibrated against Pullulan standards before measurements. FTIR results were measured on a Spectrum Two™ FT-IR spectrometer (PerkinElmer, US).

pKa measurement of phenylboronic acid molecules: The spectrophotometric method is based on the UV absorption changes, in the region 230–280 nm.[1] Each compound was dissolved in an acidic PBS standard solution and titrated with dilute NaOH. The absorption spectra were measured over the appropriate titration pH range, and an appropriate absorbance wavelength was chosen for each compound. The absorbance at that wavelength was plotted as a function of pH, and the pKa was taken as the mid-point of the titration curve (Figure S1b).[2] UV–vis absorption was conducted on Agilent Cary 8454 UV-vis spectrometer, using half-micro quartz cuvettes (Portmann Instruments). Transmittance at the wavelength of 253 nm was used for data analysis.

Swelling ratio test: The swelling ratio (SR) of the hydrogel samples in their hydrated state was evaluated using a methodology described in a previous report.[3] In this procedure, the hydrogel samples were immersed in 500 μL of cell culture medium within sealed vials, and the temperature was maintained at a constant 37°C. After a specific period, the samples were removed from the solution, any excess surface medium was blotted away with filter paper, and the hydrogels were weighed. The swelling ratio (SR) was calculated using the formula: SR (%) = (Wt - Wi) / Wi * 100%, where Wi is the initial weight, and Wt is the weight at the specified time.

Rheological measurements: All the rheology data of hydrogel samples were characterized by Malvern Instruments Kinexus equipped with a parallel plate of 8 mm diameter. The measurement was conducted at room temperature with a gap of 1 mm. A solvent trap was used to prevent the disc from drying out during the measurements.

The rupture point of a hydrogel was initially determined using an oscillatory amplitude sweep test, with a strain range from 1–200% at a constant frequency of 1 Hz (Figure S3d). For subsequent rheological testing, the selected strain must be below the rupture point.

The frequency sweep test characterizes how chemical parameters affect the mechanical properties of hydrogels. The data were analyzed using an oscillatory frequency sweep strain-controlled test with 1% strain. Storage modulus (G') and loss modulus (G'') were recorded to represent the mechanical properties of the hydrogels.

A time sweep test was conducted to assess whether hbPG-CHO undergoes self-gelation due to interactions between aldehyde and hydroxyl groups (Figure S3a) and to examine the gelation behavior of the hydrogel at different pH levels (Figure S3b). To investigate aldehyde-hydroxyl interactions, all polymers were dissolved in DMEM at a concentration of 10 wt%. For the gelation behavior study, hbPG-CHO-20 and lPG-10Bor-1F were dissolved in DMEM and DPBS, with the pH adjusted to 5, 6, 7, 8.5, and 9.5. Equal volumes of hbPG-CHO-20 and lPG-10Bor-1F solutions (at the same pH levels) were mixed, and in situ time sweep tests were performed to monitor the gelation behavior of the mixtures.

Self-healing property: To demonstrate the self-healing property, a physical disruption test was conducted. Two uniform hydrogels, made by mixing equal volumes of 10% lPG-10Bor-1F and 10% hbPG-CHO-10, were each cut into two pieces. Half of each hydrogel was then combined and left at room temperature to heal. After 48 hours, the reformed hydrogel was observed and its integrity tested (Figure S3f). Quantitative self-healing was characterized using the time sweep method with a constant frequency and varying strain (1% and 200%). According to the results of the oscillatory amplitude sweep test (Figure S3d), 1% strain is below the rupture point, while 200% strain is beyond it. The self-healing behavior of the hydrogel disks was tested by performing a rheological recovery experiment at a fixed angular frequency (1 rad·s^{-1}) with alternating strain. The amplitude oscillatory strains were switched from small strain (1.0%, 3 min for each interval) to large strain (200%, 3 min for each interval), over three cycles.

The in vitro degradation test: For the in vitro degradation test, hydrogels were prepared by mixing equal volumes of 10% lPG-10Bor-1F and 2.5% hbPG-CHO-20. These hydrogels, having the same shape and mass, were immersed in 500 μL of cell culture medium (Dulbecco's Modified Eagle Medium (DMEM)) at a constant temperature of 37°C. At predetermined time points, the hydrogel samples were removed, and their modulus was measured by rheology to assess mechanical stability.

Staining and imaging of tumor spheroid: The grown tumor spheroids were firstly fixed with 4% paraformaldehyde at room temperature for 30 min, then washed with DPBS 3 times. Cell membranes were then permeabilized using PBS containing 0.5% Triton x-100 for 10 minutes. The samples were subsequently washed three times with DPBS. For staining, the samples were first incubated in 50 nM Phalloidin-iFluor 594 Reagent (Abcam, Cambridge, UK), which was prepared in PBS containing 1% BSA, at room temperature to stain the cell cytoskeletons for 30 minutes in the dark. After three washes with DPBS, nuclei were stained with 0.5 μg/mL DAPI solution for 10 minutes. Finally, the samples were washed three times with DPBS and imaged by Leica SP8 confocal microscope (Wetzlar, Germany).

Live dead assay: Using the same method for growing tumor spheroids from HeLa-GFP cells, A549 (human lung cancer), BT-474 (ER/HER2-positive human breast cancer), HT-29 (human colorectal cancer), and SK-BR-3 (HER2-positive human breast cancer) cells were encapsulated in the dynamic hydrogels and cultured to form tumor spheroids. After 4 weeks of culturing, the DMEM was removed from the top of the dynamic hydrogels. Then, 100–200 μL of staining solutions, consisting of 2 μM calcein-AM and 4 μM ethidium homodimer-1, were added to the wells and incubated for 30 minutes at room temperature, followed by washing with PBS three times. Green fluorescent protein (GFP) and bright-field (BF) images were captured using a Zeiss Axio Observer Z1 microscope.

Supporting Results

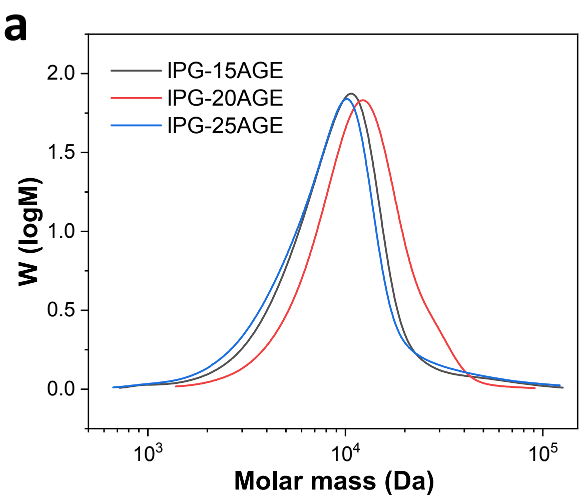

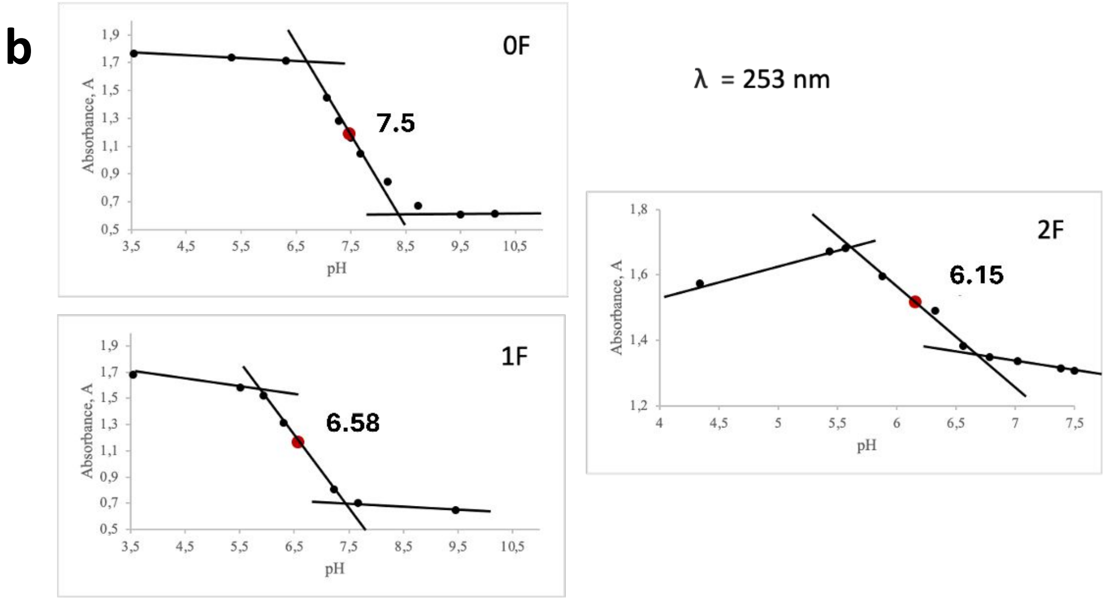

c

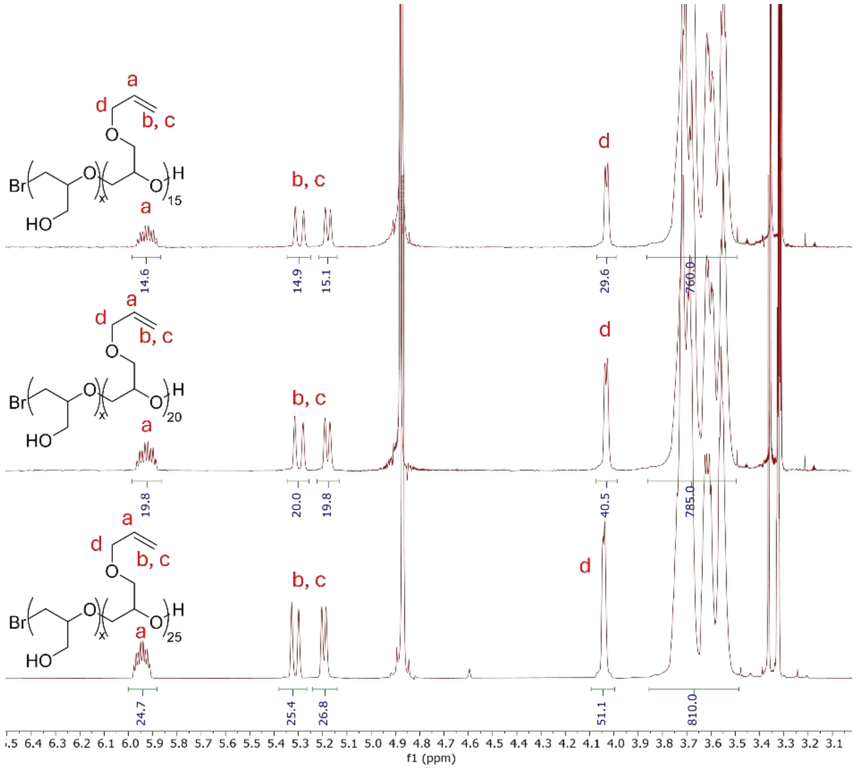

a
d
b, c
Br
O
O
O
H
x
15
HO
a
b, c
d
14.6
14.9
15.1
29.6
760.0
20
19.8
20.0
19.8
40.5
785.0
25
24.7
25.4
26.8
51.1
810.0
f1 (ppm)

d

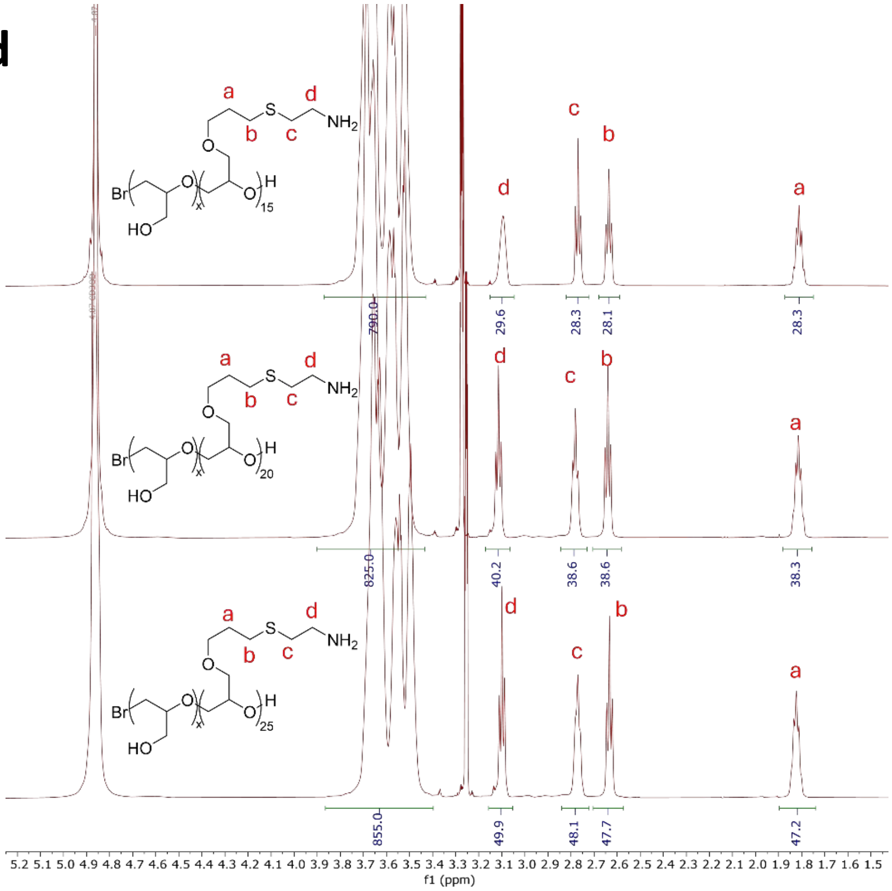

a
d
S
NH2
b
c
Br
O
O
O
H
x
15
HO
29.6
28.3
28.1
28.3
790.0
20
825.0
40.2
38.6
38.6
38.3
25
855.0
49.9
48.1
47.7
47.2
f1 (ppm)

e

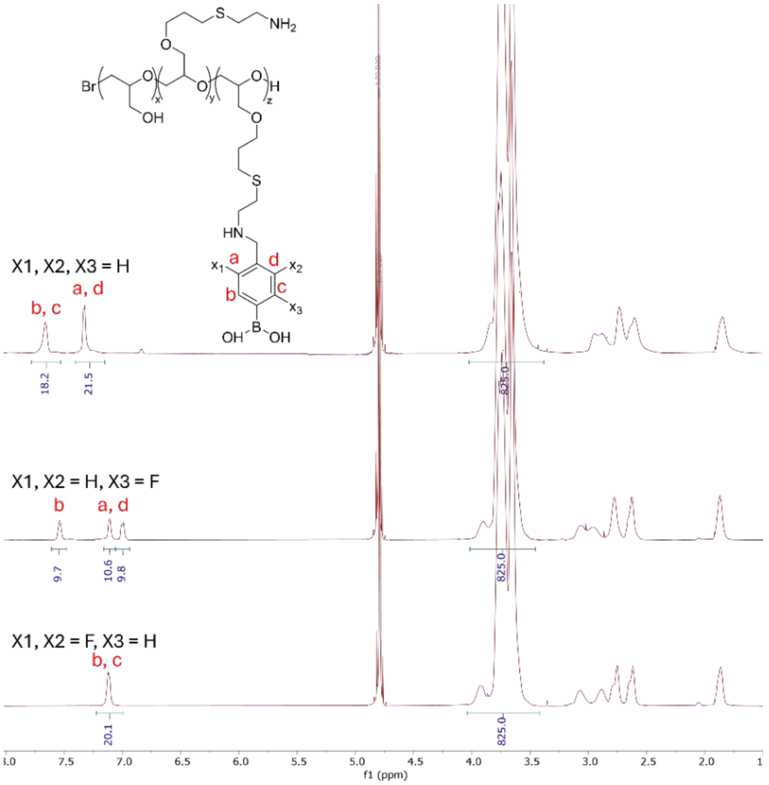

f

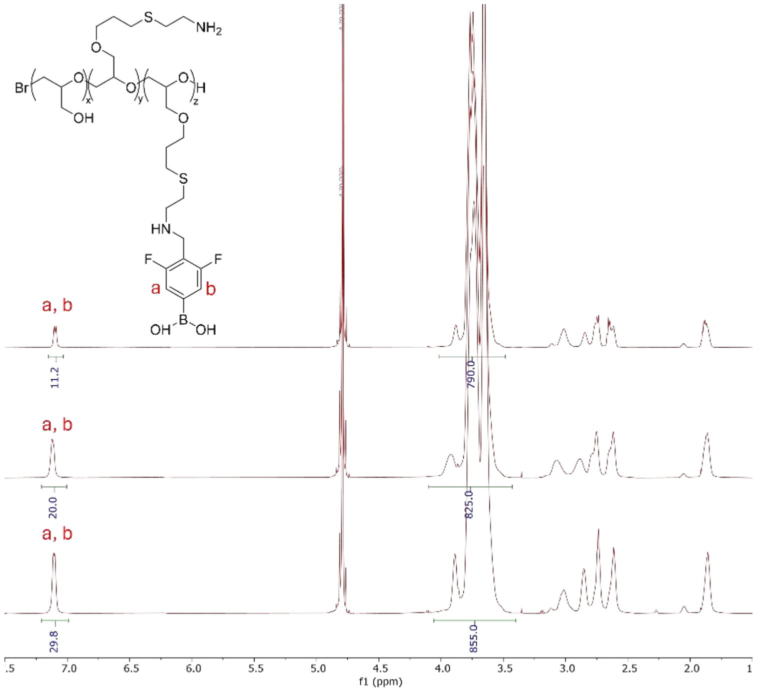

Figure S1. (a) GPC curves for lPG-15AGE, lPG-20AGE, and lPG-25AGE. (b) pH titration curves of phenylboronic acids in PBS; absorbance measured at 253 nm. (c) ^{1}H-NMR spectra (in

MeOD) of lPG-15AGE, lPG-20AGE, and lPG-25AGE. (d) ^{1}H-NMR spectra (in MeOD) of lPG-15Amine, lPG-20Amine, and lPG-25Amine. (e) ^{1}H-NMR spectra (in MeOD) of lPG functionalized with various types of phenylboronic acid. (f) ^{1}H-NMR spectra (in MeOD) of lPG functionalized with different amounts of 3,5-difluorophenylboronic acid.

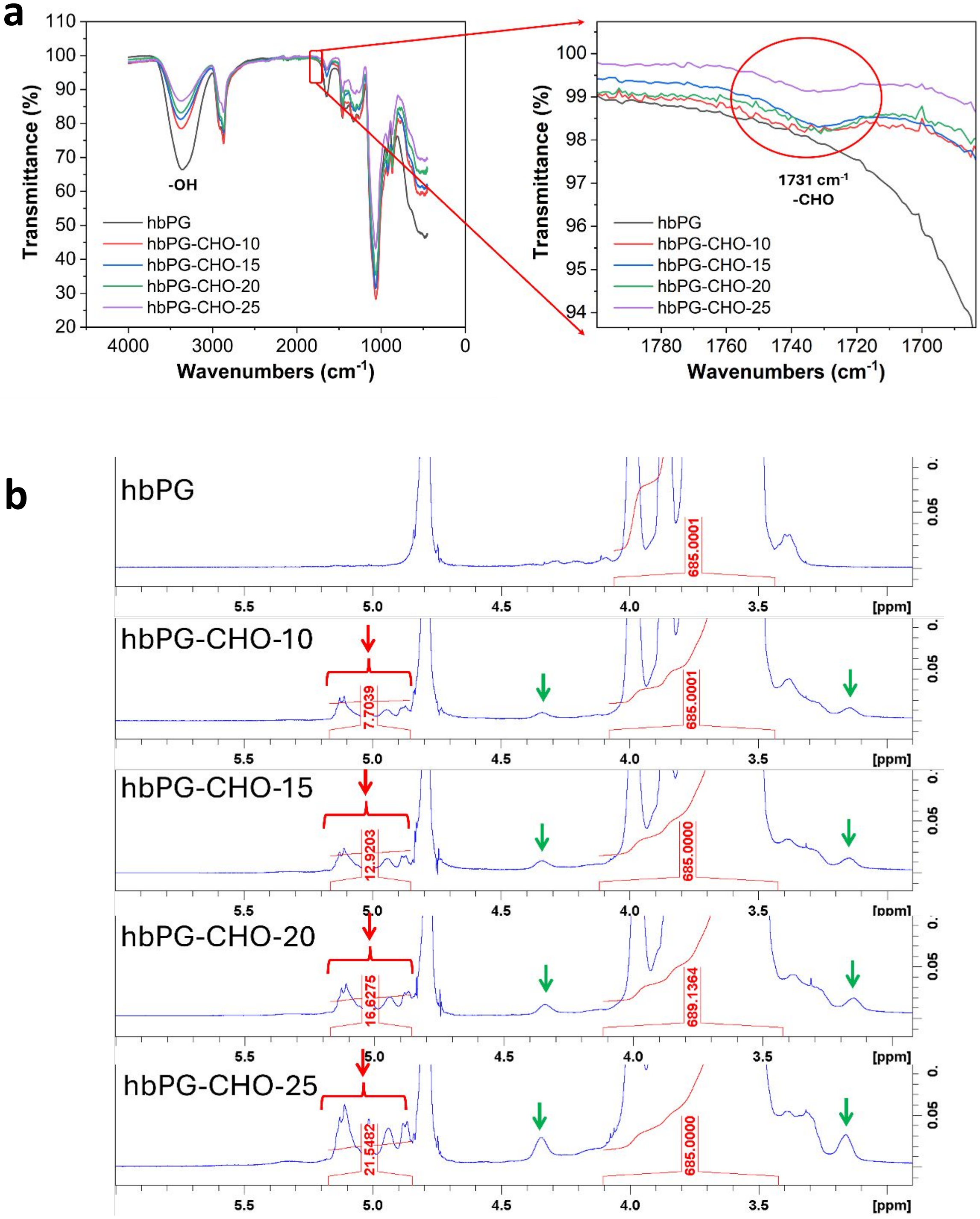

Figure S2. (a) FT-IR spectra of hbPG and hbPG-CHOs, showing a new peak at 1731 cm^{-1}, which is associated with the aldehyde bond. (b) Molecular structures of hbPG and hbPG-CHOs characterized by ^{1}H-NMR (500 MHz) using D_2O. The chemical structures of hbPG-CHOs were confirmed by the presence of characteristic signals corresponding to protons on the carbons adjacent to the aldehyde group (indicated by arrows).

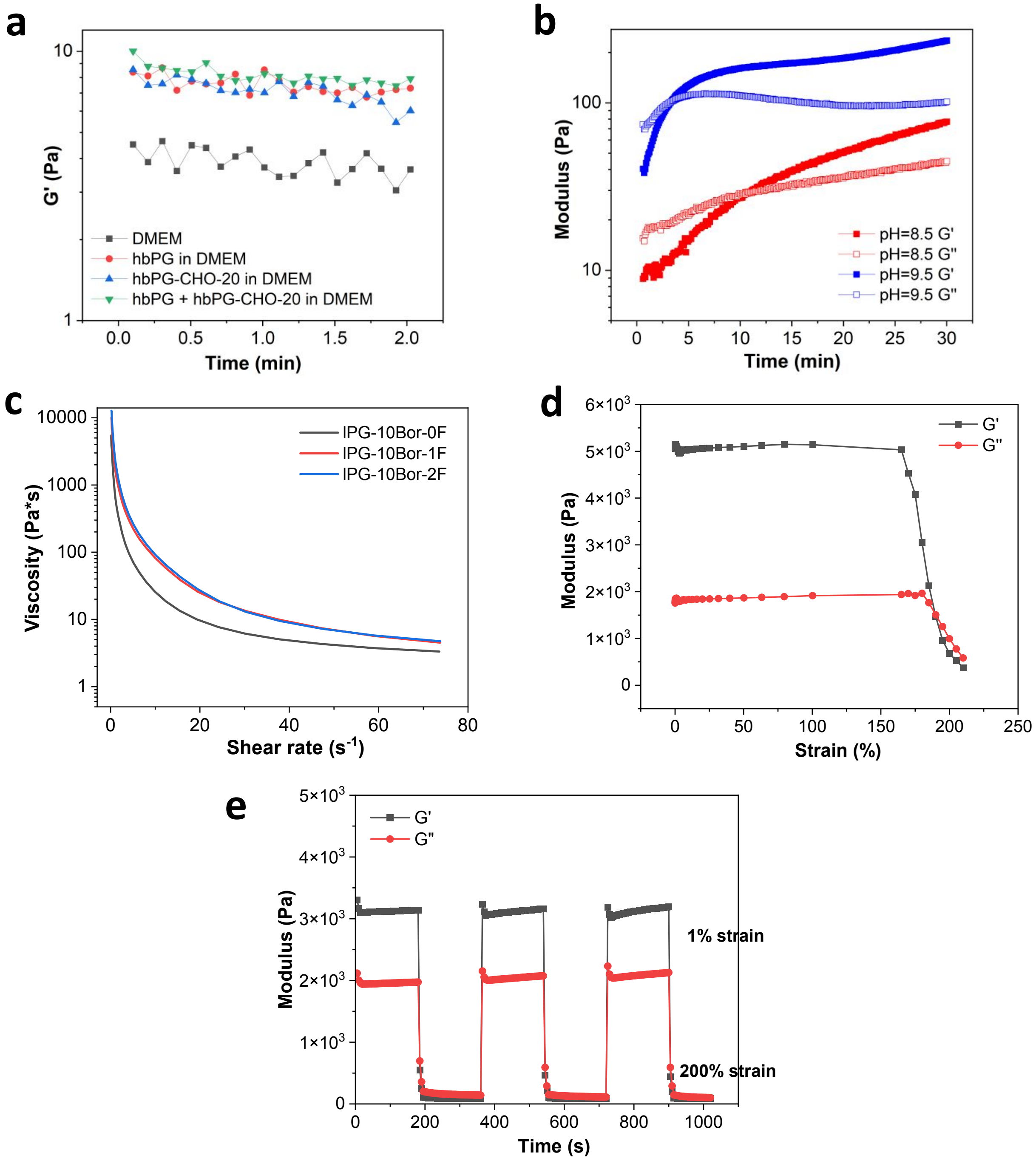

f

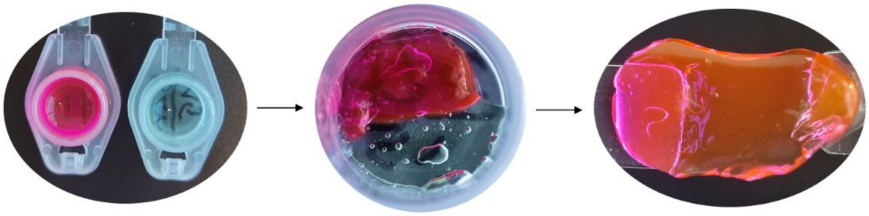

Figure S3. (a) Shear modulus of DMEM, hbPG solution, hbPG-CHO-20 solution and the mixture of hbPG and hbPG-CHO-20 measured with time sweep test. All polymers were dissolved in DMEM at 10 wt%. (b) Gelation behavior of hydrogels with different pH levels, which were measured with time sweep test. Polymer concentrations are 10 wt%. (c) Viscosity of dynamic hydrogels composed of 5 wt/v% hbPG-CHO-15 and 5 wt/v% of various phenylboronic acid-functionalized lPG types, each differing in the number of fluorine atoms on the aromatic ring. Results were obtained from flow sweep tests on a rheometer at room temperature. (d) Strain sweep test of hydrogels made from 5 wt/v% 2-fluorophenylboronic acid-functionalized lPG and 5 wt/v% hbPG-CHO-20, conducted at a frequency of 1 Hz at room temperature. (e) Shear modulus of hydrogels composed of 5 wt/v% 2-fluorophenylboronic acid-functionalized lPG and 5 wt/v% hbPG-CHO-20, measured by time sweep tests at different strain levels (1% and 200%) over various time intervals. (f) Visualization of the self-healing properties of dynamic hydrogels: first, gels formed in the lids of Eppendorf tubes (left). The gels were then cut into two pieces, and two different halves were placed together in the lid (middle). After a certain period, the two halves self-healed into a single piece (right). All concentrations mentioned above refer to the final concentrations in the dynamic hydrogels.

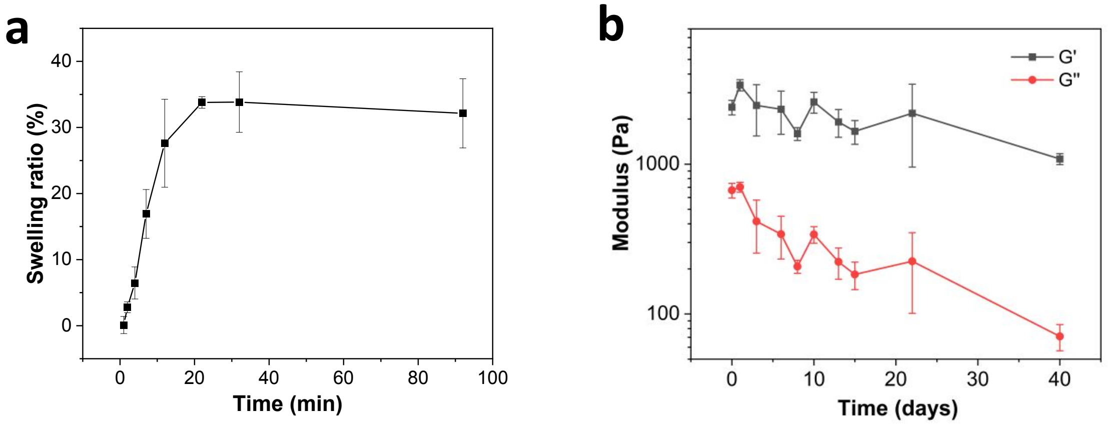

Figure S4. (a) Swelling ratio of dynamic hydrogels made from 5 wt/v% 2-fluorophenylboronic acid-functionalized lPG and 5 wt/v% hbPG-CHO-20. Data are presented as mean ± standard deviation (n = 3). (b) Shear modulus of hydrogels composed of 5 wt/v% 2-fluorophenylboronic acid-functionalized lPG and 5 wt/v% hbPG-CHO-20, immersed in cell culture medium (DMEM) at 37˚C for different durations. Results were obtained from frequency sweep tests with 1% strain and 1 Hz frequency at 25 ˚C. Data is presented as mean ± standard deviation (n = 3).

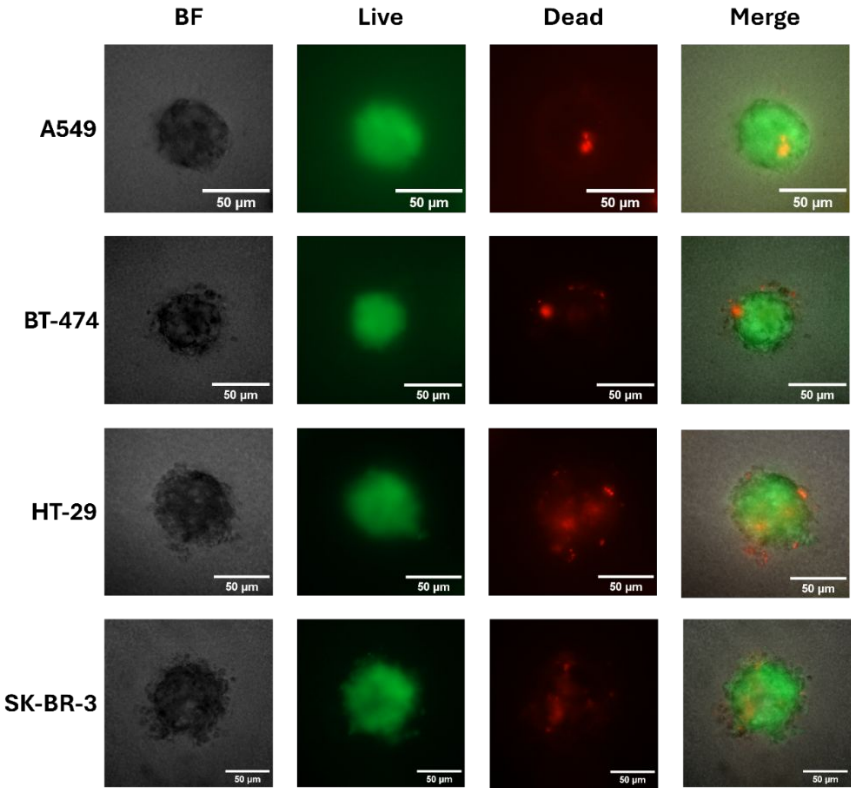

Figure S5. Images of the live/dead assay for tumor spheroids cultured in dynamic hydrogels composed of 5 wt/v% 2-fluorophenylboronic acid-functionalized lPG and 1.25 wt/v% hbPG-CHO-20 (all concentrations refer to the final concentrations in the dynamic hydrogels) over a period of 4 weeks. Green indicates live cells, while red indicates dead cells.

References:

(1) Zarzeczańska, D.; Adamczyk‐Woźniak, A.; Kulpa, A.; Ossowski, T.; Sporzyński, A. Fluorinated Boronic Acids: Acidity and Hydrolytic Stability of Fluorinated Phenylboronic Acids. *Eur. J. Inorg. Chem.* **2017,** *2017* (38-39), 4493-4498.
(2) Brooks, W. L.; Deng, C. C.; Sumerlin, B. S. Structure–Reactivity Relationships in Boronic Acid–Diol Complexation. *ACS Omega* **2018,** *3* (12), 17863-17870.
(3) Hughes, C. S.; Postovit, L. M.; Lajoie, G. A. Matrigel: A Complex Protein Mixture Required for Optimal Growth of Cell Culture. *Proteomics* **2010,** *10* (9), 1886-1890.
